# Market responses to geopolitical risk and economic policy uncertainty: Evidence from Vietnam

**DOI:** 10.1016/j.heliyon.2025.e42703

**Published:** 2025-02-14

**Authors:** Phuong Thi-Ha Cao, Duc Hong Vo

**Affiliations:** a Ho Chi Minh City Open University Vietnam, 97 Vo Van Tan Street, District 3, Ho Chi Minh City, Viet Nam; b Research Centre in Business, Economics & Resources, Ho Chi Minh City Open University Vietnam, 97 Vo Van Tan Street, District 3, Ho Chi Minh City, Viet Nam

**Keywords:** Stock return and volatility, Geopolitical risk, Economic policy uncertainty, Trade partners, TVP-VAR frequency approach, Vietnam

## Abstract

The Vietnamese stock market has traditionally been affected by local factors. However, little attention has been given to the role of external shocks, such as the geopolitical risk (GPR) and economic policy uncertainty (EPU), from Vietnam's major trade partners. Existing literature suggests that while local factors are well understood, the impact of external shocks on emerging markets such as Vietnam remains underexplored. This study investigates the effects of GPR and EPU from Vietnam's key trade partners on its stock market returns and volatility from 2000 to 2023, using the novel time-varying parameter vector autoregressive (TVP-VAR) frequency connectedness approach. Our findings reveal that EPU has a more significant effect on market volatility than GPR (42.45 per cent versus 18.80 per cent), with a long-term transmission effect for both EPU and GPR on stock returns. However, stock volatility is mainly driven by short-term transmissions. Notably, foreign EPU had a profound impact on Vietnam's stock market during the COVID-19 pandemic, whereas foreign GPR was more influential during the Russo-Ukrainian conflict. These results underscore the importance of considering external shocks when assessing stock market dynamics in Vietnam, offering valuable insights for policymakers and investors on managing risks associated with global uncertainty.

## 1 Introduction

Financial globalization over the last two decades has provided new investment opportunities to foreign investors who saw the potential portfolio diversification benefits. However, with the process of globalization and deep integration between countries, domestic financial markets, particularly those in emerging economies, have become increasingly vulnerable to external shocks [1,2]. In response to uncertainty shocks, emerging countries experience more profound and longer declines in real activity than do advanced economies [3]; [4,5]. This underscores the importance of understanding how external shocks can affect emerging financial markets, especially in the context of an increasingly interconnected global economy.

This study uses Vietnam in our analysis. Vietnam is an emerging economy that has transformed into one of the world's most rapidly growing economies [6]. This exceptional performance has attracted considerable interest and investment from both local and global investors in recent years [7]. Nevertheless, it is important to acknowledge that Vietnam is also one of the Asian countries with high trade openness, which can potentially expose its stock market to external disturbances [7]. Indeed, the extant literature has confirmed that trade is the main way by which the domestic stock markets are exposed to external shocks [8,9]. However, previous research has overlooked the crucial question of how external shocks originating from Vietnam's major trade partners impact the Vietnamese stock market in both the short and long term. Our study seeks to address this gap by investigating the spillover effect of geopolitical risk and economic policy uncertainty from Vietnam's key trade partners, which account for approximately 70 per cent of Vietnam's total trade on the Vietnamese stock market. By closely examining this issue, we expect that the study will offer valuable insights for policymakers and investors in terms of risk management.

Economic policy uncertainty (EPU) refers to the uncertainty arising from future government policies and regulatory frameworks, influencing economic conditions [10]. In contrast, geopolitical risk (GPR) refers to the uncertainty resulting from war, terrorism, and tensions that impact the stability of the global environment [11]. Previous studies have demonstrated that these economic-political uncertainties influence the stock markets. For example, Baker, Bloom, and Davis [12] that increased economic policy uncertainty is associated with higher stock market volatility, while Antonakakis, Chatziantoniou, and Filis [13] revealed that increased economic policy uncertainty is linked to lower market returns. Similarly, geopolitical risk has been shown to amplify stock market volatility [14] and negatively affect stock market returns [15].

EPU refers to uncertainty arising from potential changes in government policies and regulatory frameworks, which can significantly influence economic conditions [10]. Conversely, GPR pertains to uncertainties stemming from geopolitical events such as wars, terrorism, and international tensions that affect global stability [11]. In previous research, both EPU and GPR have been shown to influence stock markets. For example, Baker, Bloom, and Davis [16] found that increased EPU is associated with higher stock market volatility. Antonakakis, Chatziantoniou, and Filis [13] revealed that higher EPU corresponds to lower market returns. Similarly, geopolitical risk amplifies stock market volatility [14] and negatively affects stock market returns [15].

In this paper, we investigate how the EPU and GPR of ten key trade partners of Vietnam impact the return and volatility of the Vietnamese stock market in both the short and long term using the novel TVP-VAR frequency-connectedness methodology. Our findings reveal that EPU exerts a more substantial impact on the volatility of the Vietnamese stock market than the GPR. Furthermore, the effect of EPU and GPR on Vietnamese stock returns is predominantly driven by long-term transmission channels, whereas short-term transmission is the primary determinant of stock volatility. Finally, from a dynamic perspective, foreign EPU significantly influenced the Vietnamese stock market during the COVID-19 pandemic, whereas foreign GPR played a more substantial role during the Russo-Ukrainian conflict.

Our study contributes to the existing body of knowledge in several ways. First, previous studies have largely neglected a comparative analysis of the effects of external shocks on the Vietnamese stock market in both the short and long term. This motivates our study to fill this gap. Second, while much of the current literature has focused on Vietnamese stock market returns, it has overlooked the volatility dimension, which is critical for risk management and decision-making. Third, existing studies have primarily examined shocks from the US, neglecting the impact of shocks from other major trade partners of Vietnam, such as Australia, Canada, China, Japan, Germany, India, South Korea, and the UK. By incorporating these trade partners, this study provides a more comprehensive understanding of how global economic and political uncertainties influence the Vietnamese stock market, which is crucial for investors and policymakers. For investors, these empirical findings can be used to support more informed decisions on portfolio diversification and risk management, especially in times of heightened global uncertainty. For policymakers, the findings provide insights into how external factors may influence domestic financial stability and inform the development of strategies to mitigate the adverse effects of such shocks.

Following this introduction, the remainder of this paper is structured as follows. Section 2 discusses and synthesizes the literature review. Section 3 presents the data and methodology. The empirical findings are presented and discussed in section 4, followed by the concluding remarks and implications in section 5.

## 2 Literature review

From a theoretical perspective, uncertainty has a detrimental influence on economic activity [[17], [18], [19]]. Bernanke [17] indicates that businesses tend to postpone their investment needs while households tighten their spending due to the current uncertain state of the economy. Besides, uncertainty on the supply side raises the cost of employing personnel, which lowers the firm's productivity [18,20]. As such, the stock market and business circumstances may be impacted by such a negative impact from economic activity [21]. Studies indicate that macroeconomic uncertainty's effect on stock prices comes from adjustments in the required rate of returns [22,23] or changes in expected future dividends [24]. Such unfavourable effects can reduce corporate prospects and thus reduce stock market performance [12].

The economic policy uncertainty (EPU) and geopolitical risk (GPR) of major trade partners have been documented to exert adverse effects on the domestic stock market. For example, Chen and Chiang [25] discovered evidence that a rise in economic policy uncertainty (EPU) led to a fall in stock returns while investigating the Chinese stock market; nevertheless, a positive coefficient was detected in the lagged EPU as stock prices recovered. This dynamic also holds true for growth in uncertainty innovations in fiscal, monetary, trade, and global policies. The findings suggest that policy uncertainty premiums should be factored into China's stock market. An increase in US policy uncertainty negatively impacts Chinese stocks, regardless of whether the enterprises are officially owned or listed on the US market. Likewise, Tran and Vo [26] utilize the nonlinear autoregressive distributed lag approach (ARDL) to examine the effects of EPU and GPR, and US stock market volatility (VIX) on market return and volatility in the 11 Asia-Pacific countries from 1985 to 2022. The authors argue that US uncertainty indices, such as US GPR and US EPU, considerably impact Asia-Pacific stock markets. However, local GPR has very minor impacts. The authors also documented that the US EPU has a greater impact than the US GPR. Das et al. [2] have also investigated the effect of international economic policy uncertainty and geopolitical risk on the 24 emerging stock markets to determine how susceptible these markets are to different macroeconomic shocks originating in the US. Utilizing monthly data ranging from January 1997 to May 2018 and the nonparametric causality-in-quantiles test as the methodological approach, the authors found that the impact of these shocks is heterogeneous across the markets in terms of causality and intensity. Additionally, compared to GPR, the results showed that the influence of EPU is primarily substantial and important. In another study, Kannadhasan and Das [27] applied the quantile regression approach to analyze and contrast the impact of economic policy uncertainty and geopolitical risk-related shocks on nine Asian emerging stock markets. The authors discovered that EPU has a constant negative connection across all quantiles, whereas GPR is negative in the lower quantiles and positive in the intermediate and upper quantiles. The findings also demonstrated that EPU has a bigger negative impact than GPR, and the relationship between EPU and GPR on stock returns is asymmetric.

However, selected studies also provide evidence suggesting that external shocks, such as economic policy uncertainty or geopolitical risk, do not significantly impact domestic stock markets. Specifically, utilizing data from 26 emerging stock markets, Arkol and Azimli [28] find that policy uncertainty factors do not significantly explain the average returns of portfolios constructed based on well-known firm characteristics, such as size, book-to-market ratio, profitability, and investment. The authors contend that the informational content of news-based factors is already incorporated within the Fama and French risk factors. Interestingly, Zaremba et al. [29] provide evidence that emerging countries experiencing the greatest increase in geopolitical uncertainty outperform those with the smallest change by as much as 1 per cent per month. The authors attribute this anomaly to investor overreaction to geopolitical news driven by availability bias.

Several methods have been employed to examine the relationship between economic policy uncertainty (EPU), geopolitical risk (GPR), and stock market dynamics. For instance, Choi [30], Xiuwen Chen et al. [31], and Azimli and Kalmaz [32] utilize wavelet methods to investigate the co-movements between EPU, GPR indices, and financial market volatility. Additionally, quantile regression [33]; [27], the nonlinear autoregressive distributed lag (ARDL) approach [26], and quantile vector autoregression (QVAR) [34] have been applied to explore these relationships. However, the time-varying parameter vector autoregression (TVP-VAR) approach remains underutilized in this context. In this study, we employ the TVP-VAR method to analyze the impact of shocks from economic policy and geopolitical risk on the Vietnamese stock market. Compared to other approaches, the TVP-VAR method's primary advantage lies in its ability to capture dynamic changes in the relationships between variables over time. Moreover, TVP-VAR generally yields more accurate forecasts in volatile and uncertain environments, making it particularly useful for forecasting during periods of economic crises or after significant policy shifts.

Overall, studies have concentrated on the impact of uncertainties from the United States or Global on the domestic stock market. No research has explored the influence of uncertainties arising from other important trade partners. Furthermore, as a rapidly developing economy with a high degree of trade integration, particularly with China and the U.S., the Vietnamese stock market could be more susceptible to changes in EPU and GPR from these key partners. Vietnam's domestic economic policies and the growing significance of foreign investment make it an interesting context for studying how external uncertainties propagate into the stock market in both the short and long term, which is almost neglected in previous studies. Lastly, existing research has primarily focused on Vietnamese stock market returns while disregarding the associated risk dimension—volatility. Consequently, this study aims to address these gaps in the literature.

## 3 Data and methodology

### 3.1 Data

This paper examines the spillover effects of economic policy uncertainty and geopolitical risk across ten key trade partners of Vietnam on the return and volatility of the Vietnamese stock market. These ten key trade partners are Australia, Canada, China, Japan, Germany, India, South Korea, Russia, the UK, and the USA, which accounted for approximately 70 per cent of Vietnam's total import and export turnover in 2022, according to the General Statistics Office of Vietnam. In this study, geopolitical risk is measured by the GPR index proposed by Ref. [11], and economic policy uncertainty (EPU) is proxied by the economic policy uncertainty index proposed by Ref. [16]. The sample covers the period from the date when the Vietnamese stock market was established, August 2000 to June 2023. Table 1 below describes the details of each variable used in this study, while Table 2 summarizes descriptive statistics for variables used in our analysis.

**Table 1:** The variables used in this study and their data sources.

Variables	Definition	Sources
Vietnamese stock market indices	Monthly closing price of the VN Index	Thomson Reuters
Geopolitical risk index	Monthly geopolitical risk index of 10 Vietnam's key trade partners.	matteoiacoviello.com
Economic policy uncertainty	Monthly economic policy uncertainty index of 10 Vietnam's key trade partners.	policyuncertainty.com

**Table 2:** The descriptive statistics.

Variables	Observations	Mean	Std. Dev	Min	Max
EPU-AUS	275	4.55	0.54	3.24	5.82
EPU-CAN	275	5.04	0.63	3.69	6.52
EPU-CHN	275	5.08	0.77	3.24	6.74
EPU-GER	275	5.00	0.62	3.34	6.73
EPU-IND	275	4.39	0.48	3.15	5.64
EPU-JAP	275	4.63	0.28	3.85	5.47
EPU-KOR	275	4.90	0.46	3.61	6.28
EPU-RUS	275	4.93	0.78	2.51	6.87
EPU-UK	275	5.16	0.70	3.36	7.04
EPU-US	275	4.85	0.42	3.80	6.22
GPR -AUS	275	0.10	0.07	0.01	0.53
GPR-CAN	275	0.22	0.17	0.06	1.72
GPR-CHN	275	0.56	0.31	0.16	2.57
GPR -IND	275	0.22	0.13	0.06	0.95
GPR -JAP	275	0.24	0.17	0.06	1.24
GPR-KOR	275	0.32	0.25	0.06	1.81
GPR-RUS	275	0.85	0.84	0.22	8.98
GPR -UK	275	1.13	0.71	0.40	5.99
GPR-US	275	2.45	1.37	0.82	13.23
VN Index	275	609.48	343.32	103.38	1508.55

Note: **EPU** is the economic policy uncertainty. **GPR** is the geopolitical risk index. **AUS:** Australia, **CAN:** Canada, **CHN:** China, **GER:** Germany, **IND:** India, **JAP:** Japan, **KOR:** Korea, **RUS:** Russia, **UK:** the United Kingdom, **US:** the United States of America, **VN Index:** Vietnamese stock index.

### 3.2 The methodology

In this paper, Vietnamese stock market returns at month t, *LRET*_*t*_*,* are calculated as follows:

$${LRET}_{t}=\ln\left(\frac{P_{t}}{P_{t-1}}\right)$$

where *P*_*t*_ is the closing price for the month *t*, meanwhile, the Vietnamese stock market volatility at month t, *LVOL*_*t*_*,* is estimated by the Threshold GARCH (TGARCH) model. The TGARCH model, also known as the GJR model, is a popular and frequently used asymmetric model in finance. It is used to measure and handle possible asymmetries, such as leverage effects documented by Black (1976). The leverage effect arises from the observation that losses have a more pronounced impact on future volatilities than gains. This phenomenon is known as asymmetry. Specifically, the distribution of losses in this model has a heavier tail than the distribution of gains, indicating the asymmetrical nature of the model in capturing the different effects of positive and negative news on volatility. The model was developed by Zakoian (1994) and was then further developed by Glosten, Jagannathan, and Runkle (1993), who referred to it as the Glosten-Jagannathan-Runkle GARCH (GJR-GARCH) model. In the TGARCH (1,1) model, the variance equation is defined as follows.

$$h_{t}^{2}=\alpha_{0}+\alpha_{1}\varepsilon_{t-1}^{2}+\gamma D_{t-1}\varepsilon_{t-1}^{2}+\beta_{1}h_{t-1}^{2}$$

where $D_{t-1}$ is the dummy variable to account for the leverage effect and

$$D_{t-1}=\left\{ \begin{array}{c} 1.\varepsilon_{t-1}<0\;if\;bad\;news\\ 0.\varepsilon_{t-1}>0\;if\;good\;news \end{array}\right.$$

In the threshold GARCH (TGARCH) model, the leverage effect parameter, denoted as γ, plays a crucial role. When γ equals 0, the model assumes a general GARCH (p, q) form. However, when γ is a positive and significant value, the impact of bad news on conditional volatility (h2t) becomes greater than the impact of good news. Specifically, the impact of good news on volatility is represented by the parameter a1, while the impact of bad news on volatility is a1+γ. This characteristic of the TGARCH model highlights how asymmetries, such as the leverage effect, are considered, capturing the differential impact of positive and negative news on volatility. The Augmented Dickey-Fuller test (ADF) and Phillips–Perron test (PP) are employed to analyze whether a given time series is stationary to test the unit root of each variable. Table 3 indicates that all variables used in our empirical analysis are stationary.

**Table 3:** The results for the augmented Dickey-Fuller test (ADF) and Phillips–Perron test (PP).

Variables	Augmented Dickey-Fuller test	Phillips-Perron test
	Level	Level
EPU-AUS	−6.384∗∗∗	−6.915∗∗∗
EPU-CAN	−4.034∗∗∗	−6.292∗∗∗
EPU-CHN	−3.746∗∗∗	−6.818∗∗∗
EPU-GER	−4.979∗∗∗	−7.584∗∗∗
EPU-IND	−7.232∗∗∗	−7.228∗∗∗
EPU-JAP	−5.840∗∗∗	−6.016∗∗∗
EPU-KOR	−6.437∗∗∗	−7.423∗∗∗
EPU-RUS	−6.698∗∗∗	−12.818∗∗∗
EPU-UK	−4.053∗∗∗	−5.714∗∗∗
EPU-US	−5.880∗∗∗	−6.799∗∗∗
GPR -AUS	−8.570∗∗∗	−8.803∗∗∗
GPR-CAN	−7.542∗∗∗	−7.619∗∗∗
GPR-CHN	−6.630∗∗∗	−8.664∗∗∗
GPR-GER	−6.320∗∗∗	−6.661∗∗∗
GPR -IND	−8.615∗∗∗	−8.822∗∗∗
GPR -JAP	−9.013∗∗∗	−8.997∗∗∗
GPR-KOR	−6.903∗∗∗	−6.914∗∗∗
GPR-RUS	−4.969∗∗∗	−5.640∗∗∗
GPR -UK	−6.976∗∗∗	−7.220∗∗∗
GPR-US	−6.495∗∗∗	−6.670∗∗∗
LRET-VNIndex	−13.467∗∗∗	−11.996∗∗∗
LVOL-VNIndex	−5.217∗∗∗	−5.788∗∗∗

Note: ∗∗∗ is significant at 1 per cent. EPU is the economic policy uncertainty. GPR is the geopolitical risk index. **AUS:** Australia, **CAN:** Canada, **CHN:** China, **GER:** Germany, **IND:** India, **JAP:** Japan, **KOR:** Korea, **RUS:** Russia, **UK:** the United Kingdom, **US:** the United States of America, **LRET-VN Index:** Returns of Vietnamese stock index. **LVOL-VNIndex**: Volatility of Vietnamese stock index.

Next, we analyze the moment-based risk connectedness from uncertainty to the Vietnamese stock market using the TVP-VAR-based frequency connectedness approach. This approach is based on Antonakakis, Chatziantoniou, and Gabauer [35] work.

## 4 Empirical results

### 4.1 Spillovers from EPU and GPR of 10 countries to Vietnamese stock market returns

Table 4 presents static evidence of spillovers from EPU and GPR of key trade partners to Vietnamese stock market returns in the total period, short-run and long-run. In general, the spillover effects of EPU and GPR on Vietnamese stock returns are relatively substantial, with the spillover effect of GPR being more pronounced than that of EPU. The directional spillover FROM other EPUs of Vietnam's key trade partners contributed to the returns on the Vietnamese stock market of 20.61 %, implying that on average, 20.61 % of the forecast error variance in Vietnamese stock returns is captured by the EPU of these countries, with the remaining 79.39 % being captured by the peculiarities of the Vietnamese stock market. Meanwhile, the GPR of the key trade partners contributed to the 29.6 % returns on the Vietnamese stock market. From the perspective of frequency decomposition, the impact of EPU on Vietnamese stock returns is mainly driven by transmission in the long term (12.19 %) rather than that in the short term (8.42 %). In contrast, the short-term and long-term transmission of GPR to Vietnamese stock returns are equivalent (14.77 % and 14.88 %, respectively). These results can be explained by the view that geopolitical risks often involve unexpected events, which can disrupt international trade, change investment flows, or increase global instability. These events could cause an immediate reaction from global investors, leading to a flight from emerging markets, including Vietnam and a shift to safer assets such as gold or government bonds. As such, stock market returns are strongly affected in the short term. Meanwhile, EPU is frequently linked to fiscal, monetary, or tax policy changes, which may either be anticipated or managed through signals provided by governments and central banks. These policy changes often take time to be reflected in stock prices, as investors typically await the actual implementation of these policies. However, investors can evaluate their potential impact over time and adjust their investment strategies incrementally.

**Table 4:** The static spillover from EPU and GPR of 10 countries to Vietnamese stock market returns.

Panel A: Spillovers from EPU of Vietnam's 10 key trade partners to Vietnamese stock market returns			
EPU	Total	Short-run	Long-run
AUS	2.36	0.50	1.86
CAN	1.82	0.57	1.25
CHN	1.71	0.86	0.86
GER	2.18	1.00	1.18
IND	1.80	0.97	0.83
JAP	3.80	1.16	2.64
KOR	1.98	0.98	1.00
RUS	1.40	1.09	0.30
UK	2.14	0.73	1.42
US	1.40	0.55	0.85
From other EPU to Vietnamese stock market returns	20.61	8.42	12.19

**Panel B: Spillovers from GPR of Vietnam's 10 key trade partners to Vietnamese stock market returns**			
**GPR**	**Total**	**Short-run**	**Long-run**

AUS	2.90	1.21	1.68
CAN	1.95	1.37	0.57
CHN	2.06	0.70	1.36
GER	3.69	1.60	2.09
IND	2.89	2.14	0.74
JAP	3.60	1.48	2.12
KOR	3.80	1.37	2.43
RUS	2.78	1.39	1.39
UK	3.29	1.90	1.38
US	2.67	1.57	1.10
From other GPR to Vietnamese stock market returns	29.62	14.74	14.88

Note: EPU is the economic policy uncertainty. GPR is the geopolitical risk index. AUS: Australia, CAN: Canada, CHN: China, GER: Germany, IND: India, JAP: Japan, KOR: Korea, RUS: Russia, UK: the United Kingdom, US: the United States of America.

In light of the pairwise directional connectedness, it is noticeable that the Japanese EPU is the highest contributor to Vietnamese stock market returns, contributing 3.80 %, followed by Australia (2.36 %), Germany (2.18 %), the UK (2.14 %) and Korea (1.98 %), respectively. Moreover, the Japanese EPU demonstrates consistent patterns because of its highest contribution to the returns of the Vietnamese stock market in the short and long term. This consistent influence underscores the significant impact of Japan's economic policies on Vietnam's financial market, likely due to the strong trade and economic ties between the two nations. It is also important to highlight that India's GPR is the most significant contributor to Vietnam's stock returns in the short term, contributing 2.14 %. In contrast, Korea's GPR emerges as the dominant contributor in the long term, with a contribution of 2.43 %. This shift in the impact of GPR from India to Korea over time reflects the changing nature of geopolitical risk and its varying effects on the Vietnamese market depending on the time horizon.

**Fig. 1:**
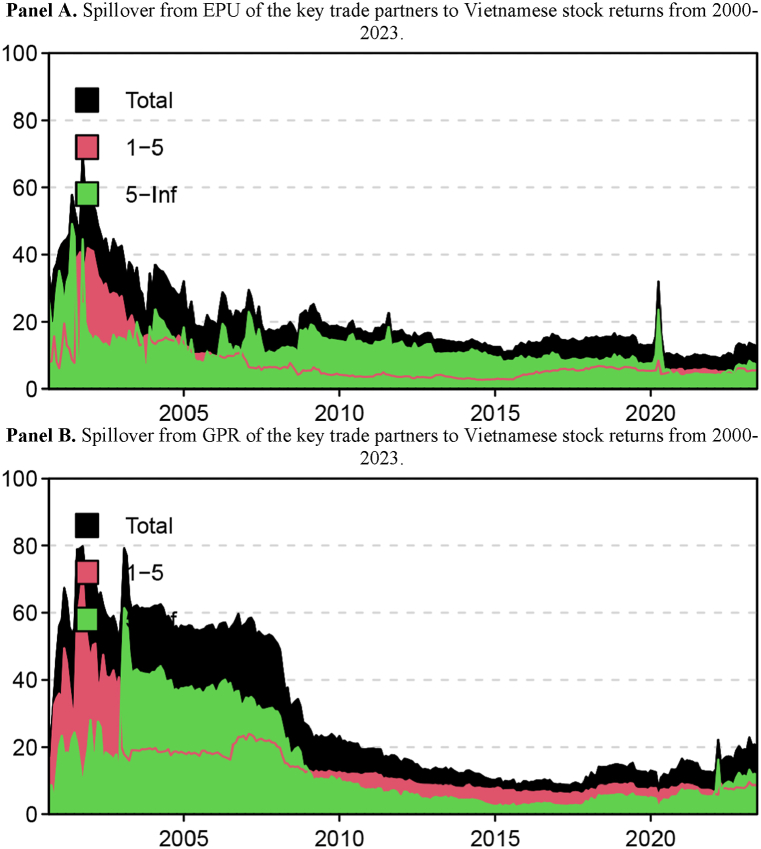
Spillover from EPU and GPR of Vietnam's key trade partners to Vietnamese stock market return from 2000 to 2023. Note: The red-shaded area represents spillover in the short term, while the green-shaded area represents in the long term.

The results presented in Table 4 focus solely on the return spillover of EPU and GPR throughout the analyzed period, disregarding any changes in the spillover indices over time. Therefore, we proceeded to investigate the variation in spillover from EPU and GPR of key trade partners to Vietnamese stock market returns for the period 2000–2023, as displayed in Fig. 1, which demonstrates not only the total connectedness from EPU and GPR (black-shaded area) but also the decomposition into components in the short-run (red-shaded area) and the long-run (green-shaded area). The dynamic connectedness index from EPU and GPR of ten countries to Vietnamese stock returns fluctuated over time, ranging from 8.82 per cent to 70.52 per cent for the EPU and from 7.28 per cent to 79.74 per cent for GPR, which implies that the degree of transmission from EPU and GPR of ten countries to Vietnamese stock returns in this system strongly varied with time. In addition, the transmission from EPU varied from 2.66 per cent to 41.92 per cent in the short term and from 3.64 per cent to 49.04 per cent in the long run. In the meantime, the GPR transmission fluctuated between 5.46 and 71.60 per cent over the short term and between 1.82 and 61.35 per cent over the long term. Once again, the frequency decomposition results show that the primary source of the spillovers is the long-term components of both GPR and EPU. The magnitude of short-term and long-term spillovers varies over time. Specifically, the short-term components of EPU were significantly larger than the long-term components from 2002 to 2003. However, from 2021 to 2022, the short-term components of EPU were only slightly higher than the long-term components. Similarly, from 2000 to 2002, the short-term components of GPR were considerably greater than the long-term components. Nonetheless, from 2009 to 2021, the short-term components of GPR were only slightly higher.

Fig. 1 also shows that the transmission dynamics from the Geopolitical Risk (GPR) of key trade partners to Vietnamese stock returns reached their peak during the years 2001–2002. This coincided with the September 11, 2001, terrorist attacks in the United States, which had far-reaching impacts on the global political and economic systems. It is important to note, however, that the evidence suggests that the spillover effects of foreign Economic Policy Uncertainty (EPU) on Vietnamese stock market returns during the September 11 event were significantly lower (around 60 %) compared to the spillover effects of foreign GPRs (around 90 %). This difference is understandable as GPR captures risks related to war and terrorism, which EPU does not encompass. In contrast, during the 2008 global financial crisis, the spillover effect of foreign EPUs on Vietnamese stock market returns reached nearly 35 %, whereas the spillover effect of foreign GPRs was only 22 %. This indicates that the 2008 global financial crisis was primarily an economic event rather than a political one. These findings suggest that GPR and EPU cannot be perfectly substituted for each other and should be considered separately to capture the two types of uncertainties.

Furthermore, the returns of the Vietnamese stock market have exhibited heightened vulnerability to external Economic Policy Uncertainty (EPU) during the onset of the COVID-19 pandemic, as well as increased sensitivity to external Geopolitical Risk (GPR) during the eruption of the Russia-Ukraine conflict. Previous studies have demonstrated that Black Swan events, such as the COVID-19 pandemic and the Russia-Ukraine conflict, have exerted profound impacts on stock market performance [36]; [[37], [38], [39]]. Another noteworthy aspect is that the Vietnamese stock market appears to be susceptible to external shocks during its early stages of development when it has not fully matured. However, in recent years, there has been a notable decrease in the magnitude of spillover effects from external shocks compared to the initial years of establishment. These can be explained by the maturation of the Vietnamese stock market, improved market infrastructure, stronger regulatory frameworks, increased economic integration, and a more confident and diversified investor base. These factors have collectively reduced the market's susceptibility to external shocks. These can be explained by the young nature of the Vietnamese stock market, improved market infrastructure, stronger regulatory frameworks, increased economic integration, and a more confident and diversified investor base. Collectively, these factors have reduced the market's susceptibility to external shocks.

### 4.2 Spillover from the economic policy uncertainty (EPU) and the geopolitical risk (GPR) of Vietnam's 10 trading partners to Vietnamese stock market volatility

Spillover from EPU and GPR of key trade partners to Vietnamese stock market volatility is illustrated in Table 5. The results demonstrate that the EPU and GPR in the network contributed to the volatility of the Vietnamese stock market of 42.45 % and 18.80 %, respectively. This finding aligns with the signal precision theory of Pastor and Veronesi [40], which posits that market volatility is influenced by the interaction between political uncertainty and the precision of the signals available to investors. Uncertainty signals stemming from government policies, such as fiscal policies, regulations, and taxation, typically emerge from well-established formal institutions. Such uncertainty can have a prolonged impact on stock market volatility, as it shapes investor confidence regarding the future trajectory of the economy and financial markets. In contrast, signals linked to rare events, such as terrorist attacks or wars, are often random and harder for investors to interpret. As a result, markets tend to exhibit more significant reactions to Economic Policy Uncertainty (EPU), whereas they are less responsive to Geopolitical Risk (GPR). Furthermore, GPR may lead to short-term volatility spikes but does not necessarily induce long-term shifts in market expectations.

**Table 5:** Static spillover from EPU and GPR of 10 countries to Vietnamese stock market volatility.

Panel A: Spillovers from EPU of Vietnam's 10 key trade partners to Vietnamese stock market volatility			
EPU	Total	Short-run	Long-run
AUS	4.28	1.83	2.45
CAN	6.22	3.33	2.89
CHN	2.02	1.66	0.36
GER	5.53	3.68	1.85
IND	2.97	2.43	0.54
JAP	5.87	4.55	1.32
KOR	0.68	0.45	0.23
RUS	1.26	0.77	0.49
UK	3.83	1.17	2.67
US	9.78	7.39	2.39
From other EPU to Vietnamese stock market volatility	42.45	27.27	15.17

**Panel B: Spillovers from GPR of Vietnam's 10 key trade partners to Vietnamese stock market volatility**			
**GPR**	**Total**	**Short-run**	**Long-run**

AUS	1.05	0.61	0.44
CAN	2.85	1.54	1.31
CHN	1.67	0.72	0.95
GER	2.30	1.21	1.09
IND	1.60	0.56	1.04
JAP	1.64	1.08	0.56
KOR	1.56	0.87	0.69
RUS	2.20	0.83	1.36
UK	2.07	1.12	0.95
US	1.87	1.18	0.69
From other GPR to Vietnamese stock market volatility	18.80	9.72	9.08

Note: EPU is the economic policy uncertainty. GPR is the geopolitical risk index. AUS: Australia, CAN: Canada, CHN: China, GER: Germany, IND: India, JAP: Japan, KOR: Korea, RUS: Russia, UK: the United Kingdom, US: the United States of America.

From a frequency decomposition perspective, short-term transmission remains the primary driver of the impact of EPU on Vietnamese stock volatility, with a contribution of 27.27 per cent, compared to 15.17 per cent in the long term. This suggests that market participants react more immediately to international policy shifts, which can create heightened short-term volatility. Likewise, the impact of GPR on Vietnamese stock volatility is mainly driven by short-term transmission (9.72 per cent) compared to its long-term effect (9.08 per cent). This finding indicates that while impactful geopolitical risks tend to affect markets quickly in response to sudden events, they do not have the same prolonged impact on long-term market expectations as EPU.

The findings additionally demonstrate that, when compared to the EPU of the remaining nations, the US contributes the most to the volatility of the Vietnamese stock market (9.78 per cent), followed by Canada (6.22 per cent), Japan (5.87 per cent), Germany (5.53 per cent). This is indicative of the US's significant influence on the global economic environment, which can lead to immediate shifts in investor sentiment and market reactions. Meanwhile, in terms of GPR, Canada is the most significant contributor to the Vietnamese stock market volatility, accounting for 2.85 per cent, followed by Germany (2.30 per cent), Russia (2.20 per cent), the UK (2.07 per cent) and the US (1.87 per cent). Furthermore, from the point of view of frequency decomposition, transmission in the short term (27.27 per cent) rather than that in the long term (15.17 per cent) primarily drives the impact of EPU on the volatility of Vietnamese stocks. Likewise, the impact of GPR on Vietnamese stock volatility is also mainly driven by transmission in the short term (9.72 per cent) rather than that in the long term (9.08 per cent). Notably, the US EPU (7.39 per cent) mainly impacts Vietnamese stock volatility in the short term, whereas the Canadian EPU (2.89 per cent) has the greatest long-term influence. A similar situation occurs with GPR, whereby Russia's GPR (accounting for 1.36 per cent) has the greatest long-term impact on Vietnam's stock volatility. This could be due to long-standing regional geopolitical factors that affect broader economic stability and global trade. On the other hand, Canada's GPR has the most substantial short-term impact **(**accounting for 1.54 per cent), suggesting that sudden geopolitical developments in Canada can immediately affect investor sentiment and market volatility in Vietnam.

**Fig. 2:**
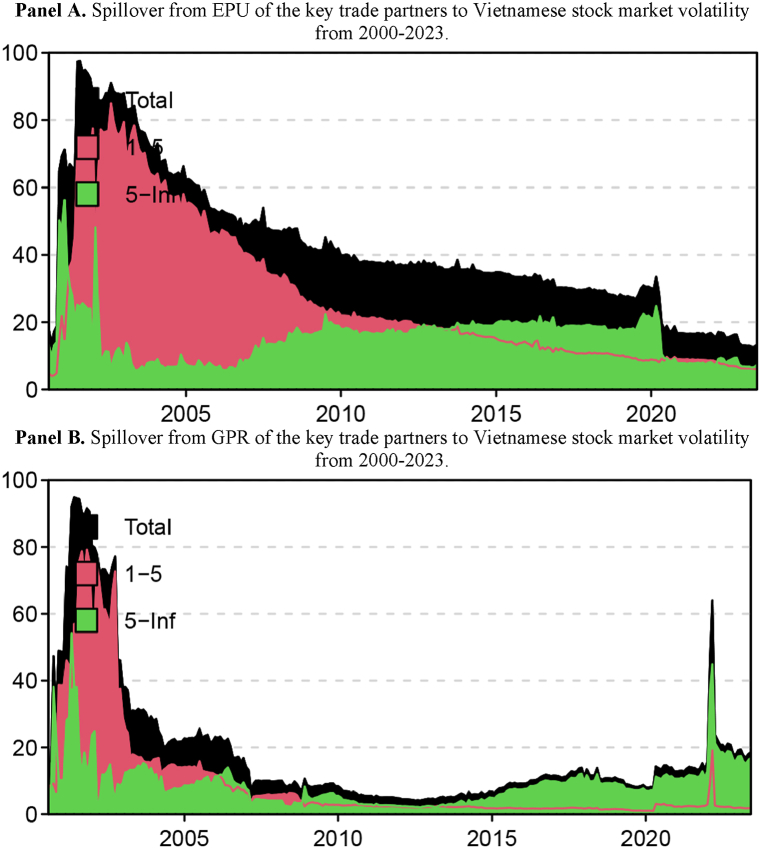
Spillover from EPU and GPR of Vietnam's key trade partners to Vietnamese stock market volatility from 2000 to 2023. Note: The red-shaded area represents spillover in the short term, while the green-shaded area represents spillover in the long term.

We then examine the dynamic spillover from EPU and GPR to Vietnamese stock market volatility from 2000 to 2023, as shown in Fig. 2. The dynamic connectedness index from EPU and GPR of key trade partners to Vietnamese stock volatility fluctuated strongly over time, ranging from 12.63 per cent to 97.54 per cent for EPU and from 4.11 per cent to 94.82 per cent for GPR, implying that the degree of transmission from EPU and GPR to Vietnamese stock volatility in this system varied over time. As shown by the frequency decomposition results, short-term transmission from EPU ranged from 3.99 per cent to 84.99 per cent, while long-term transmission varied from 5.52 per cent to 56.23 per cent. Meanwhile, the GPR transmission varied throughout the short term and long term, ranging from 1.00 to 79.13 per cent and 2.05 to 54.08 per cent, respectively. Additionally, the red regions dominate the spillover, indicating that the short-term components are the primary cause of the spillover. Vietnamese stock volatility was primarily impacted by EPU in the short term between 2001 and 2012; however, from 2013 to 2020, the long-term dominated, and after that, the short and long terms were almost equivalent. From 2001 to 2006, GPR primarily affected the volatility of Vietnamese stocks in the short term. From 2006 to 2009, however, the short- and long-term effects were nearly equal, and beginning in 2008, the long-term effects also predominated. Besides, once again, we witnessed an immediate increase in the effects of spillovers from the EPU and GPR in the system to Vietnam's stock market volatility, which peaked on the September 11 terrorist attack in 2001. This sudden surge underscores the high sensitivity of financial markets to geopolitical shocks, particularly when they involve uncertainty about global security and policy responses. Another notable spike occurred during the Russia-Ukraine conflict in early 2022, particularly in the context of GPR. This geopolitical event led to an unexpected increase in spillover effects on Vietnamese stock market volatility, as shown in Panel B of the figure. The Russia-Ukraine conflict has been identified in several recent studies as a significant political shock to global financial markets ([32]; [41]; B. C. [42,43]). The analysis suggests that the spillover from GPR, driven by this conflict, had a profound and immediate impact on market behavior, reflecting the far-reaching consequences of geopolitical disruptions.

## 5 The concluding remarks and implications

The globe has lately witnessed substantial instability induced by the US-China trade war, the COVID-19 pandemic in January 2020, the ongoing Russia-Ukraine conflict in February 2022, and the ongoing Israel-Palestine war. As a result, analyzing the influence of external shocks, such as economic policy uncertainty and geopolitical risk, on the local stock market is critical, particularly in an emerging market like Vietnam. As such, this paper investigates the effects of geopolitical risk and economic policy uncertainty from Vietnam's major trading partners on the Vietnamese stock market over the period 2000–2023 utilizing the novel TVP-VAR frequency connectedness approach.

Key empirical findings and several policy implications that have emerged based on the findings from this study can be summarized as follows. Economic policy uncertainty had a more significant impact on the volatility of the Vietnamese stock market (42.45 per cent) compared to geopolitical risk (18.80 per cent). Additionally, when considering the breakdown of frequencies, long-term transmission plays a crucial role in driving the effect of economic policy uncertainty and geopolitical risks on Vietnamese stock returns. On the other hand, short-term transmission primarily influences Vietnamese stock volatility. Furthermore, during the COVID-19 pandemic, foreign economic policy uncertainties had a notable influence on the Vietnamese stock market. In contrast, foreign geopolitical risks exerted a significant impact during the Russo-Ukrainian conflict outbreak.

Based on the findings of our study, several practical implications have emerged for policymakers, investors, and stakeholders. Given that Vietnam's stock volatility is more influenced by economic policy uncertainty from trade partners than the geopolitical risks, policymakers should focus on monitoring and responding to changes in the economic policies of key trade partners. This focus can help mitigate potential disruptions in trade and investment caused by the shifts in economic policies from foreign trade partners. For instance, if a key trade partner is expected to implement new trade tariffs, tax policies, or regulatory changes, the Vietnamese government could take proactive measures, such as engaging in diplomatic discussions to understand the changing effects of these policies on trade flows. On the other hand, while geopolitical risks, such as international conflicts or diplomatic tensions, are important, they appear to exert a lesser influence on stock market volatility in Vietnam than EPU. Policymakers, therefore, may choose to focus on addressing EPU-related challenges such as negotiating trade agreements, diversifying trade relations, and maintaining stable economic policies while still being prepared for geopolitical risks, but not necessarily allocating resources to address these risks on a priority basis as those risks are simply beyond the government's effort.

Moreover, the study reveals that the effects of EPU and GPR on the Vietnamese stock market differ across the short and long term. This insight is valuable for policymakers and investors when designing and implementing policies and strategies. Policymakers should consider the time horizon of their policy actions and their potential impacts on market stability. For example, short-term policy interventions may be more effective in addressing volatility. In contrast, long-term strategic planning, such as fostering stronger economic partnerships and aligning with global policy trends, may better support sustained growth in stock returns. Investors, on the other hand, should take these frequency differences into account when forming investment strategies. In periods of heightened EPU, they may need to adjust their portfolios to mitigate volatility by shifting investments into more defensive sectors or increasing their cash positions. In contrast, when GPR is more relevant, investors may look at hedging strategies or adjust their exposure to sectors sensitive to international risks, such as energy or export-oriented industries. Additionally, given that the influence of EPU and GPR can vary over time, investors should adopt a dynamic approach, frequently reassessing the evolving political and economic landscape of Vietnam's key trade partners. For other stakeholders, such as financial institutions or analysts, understanding the differential impacts of EPU and GPR on stock market dynamics is essential for providing informed advice to clients or making investment decisions. Institutions that focus on emerging markets like Vietnam should incorporate these insights into their risk models, especially considering the increased interconnectedness of global markets and the potential for external shocks to influence market outcomes.

While this study provides valuable insights into the impact of economic policy uncertainty and geopolitical risk on Vietnam's stock market, our study exhibits several limitations. First, the study focuses on a limited set of trade partners, which may not fully capture the global interconnectedness of Vietnam's financial market. Future research could expand the analysis by incorporating additional indices from other key trading partners or emerging markets, which might yield different or more comprehensive results. Second, our study relies on specific measures of EPU and GPR, which, while widely used, may not capture all dimensions of economic and geopolitical uncertainty. Alternative methods, such as the inclusion of new indices, such as trade policy uncertainty (TPU), could provide a more nuanced understanding of how external shocks affect market behavior.

## CRediT authorship contribution statement

**Phuong Thi-Ha Cao:** Writing – original draft, Software, Investigation, Formal analysis, Data curation, Conceptualization. **Duc Hong Vo:** Writing – review & editing, Writing – original draft, Visualization, Validation, Supervision, Software, Resources, Project administration, Methodology, Investigation, Formal analysis, Conceptualization.

## Informed consent

Not applicable.

## Ethics approval

This research did not involve any human participants or animals.

## Data availability statement

Data included in the article/supplementary material is referenced in the article.

## Funding acknowledgements

This research received no specific grant from any funding agency in the public, commercial, or not-for-profit sectors.

## Declaration of competing interest

The authors declare that they have no known competing financial interests or personal relationships that could have appeared to influence the work reported in this paper.
